# Investigating methods to enhance interpretability and performance in cardiac MRI for myocardial scarring diagnosis using convolutional neural network classification and One Match

**DOI:** 10.1371/journal.pone.0313971

**Published:** 2025-06-09

**Authors:** Michael H. Udin, Sara Armstrong, Alice Kai, Scott T. Doyle, Saraswati Pokharel, Ciprian N. Ionita, Umesh C. Sharma

**Affiliations:** 1 Department of Medicine, State University of New York at Buffalo, Buffalo, New York, United States of America; 2 Department of Biomedical Engineering, State University of New York at Buffalo, Buffalo, New York, United States of America; 3 Department of Pathology, Roswell Park Comprehensive Cancer Center, Buffalo, New York, United States of America; 4 Canon Stroke and Vascular Research Center, Buffalo, New York, United States of America; South China University of Technology, CHINA

## Abstract

Machine learning (ML) classification of myocardial scarring in cardiac MRI is often hindered by limited explainability, particularly with convolutional neural networks (CNNs). To address this, we developed One Match (OM), an algorithm that builds on template matching to improve on both the explainability and performance of ML myocardial scaring classification. By incorporating OM, we aim to foster trust in AI models for medical diagnostics and demonstrate that improved interpretability does not have to compromise classification accuracy. Using a cardiac MRI dataset from 279 patients, this study evaluates One Match, which classifies myocardial scarring in images by matching each image to a set of labeled template images. It uses the highest correlation score from these matches for classification and is compared to a traditional sequential CNN. Enhancements such as autodidactic enhancement (AE) and patient-level classifications (PLCs) were applied to improve the predictive accuracy of both methods. Results are reported as follows: accuracy, sensitivity, specificity, precision, and F1-score. The highest classification performance was observed with the OM algorithm when enhanced by both AE and PLCs, 95.3% accuracy, 92.3% sensitivity, 96.7% specificity, 92.3% precision, and 92.3% F1-score, marking a significant improvement over the base configurations. AE alone had a positive impact on OM increasing accuracy from 89.0% to 93.2%, but decreased the accuracy of the CNN from 85.3% to 82.9%. In contrast, PLCs improved accuracy for both the CNN and OM, raising the CNN’s accuracy by 4.2% and OM’s by 7.4%. This study demonstrates the effectiveness of OM in classifying myocardial scars, particularly when enhanced with AE and PLCs. The interpretability of OM also enabled the examination of misclassifications, providing insights that could accelerate development and foster greater trust among clinical stakeholders.

## Introduction

Prompt and accurate diagnosis of myocardial scarring (MS) is vital for effective patient management and treatment planning, particularly following myocardial infarctions [1]. MS is associated with an increased risk of arrhythmias and heart failure, with early detection allowing for timely intervention, which can improve patient outcomes [1–3]. Contrast-enhanced cardiac MRI (CMR) plays an important role by highlighting fibrotic and necrotic regions, which is necessary for thorough clinical assessment [4]. While the interpretation of CMR images is generally straightforward, it requires specialized knowledge and can be time-consuming. Implementing automated AI-driven tools can streamline this process, significantly reducing physician workload by handling routine diagnostic tasks. This automation not only speeds up diagnosis but also ensures that clinicians can dedicate more time to patient care and complex cases, enhancing overall healthcare efficiency.

As ongoing efforts in the imaging community seek to improve clinical efficiency through automation, convolutional neural networks (CNNs) have emerged as a reliable and widely used method for classifying pathological features such as MS [5–7]. CNNs excel by recognizing spatial patterns and learning complex hierarchical representations, achieving high-accuracy classifications that can enhance diagnostic efficiency. Despite their efficacy, CNNs have been criticized for their opacity in decision-making, which leads to challenges in explaining their predictions and optimizing their algorithms. The black-box nature of CNNs also challenges their adoption in clinical settings, where clear communication is paramount for establishing trust and fostering integration. In particular, lack of transparency can undermine trust among medical professionals, patients, and regulatory bodies, which is essential given the high stakes of clinical decisions. Comparatively, other methods like support vector machines, random forests, and recurrent neural networks have been employed for classifying pathological features but may lack performance or clarity in decision-making [8–10]. These shortcomings highlight the need for innovative AI approaches that combine the efficiency of such algorithms with greater transparency, ensuring that these tools can be trusted and optimized within clinical workflows.

In response, explainable AI (XAI) has emerged as a field focused on elucidating the decision-making processes of these complex models. Methods such as class activation maps (CAMs) [11], Local Interpretable Model-agnostic Explanations (LIME) [12], and SHapley Additive exPlanations (SHAP) [13] have been applied to CNNs to enhance their interpretability. CAMs visually highlight important areas in an image for CNN predictions, but they do not explain why these areas are significant to the model. With LIME, input data is perturbed to observe changes in model outputs, creating visualizations that highlight influential image regions but offer limited explanations for why these regions are important. Similarly, SHAP assesses feature importance by masking areas in the input data, but it also lacks explanations for the specific impact of these areas on classification outcomes. Moreover, methods like LIME and SHAP function as secondary models to approximate the original model’s behavior, compounding any inaccuracies. This increases the risk that the explanations could be misleading, undermining trust in their accuracy. While CAMs, LIME, and SHAP represent significant strides in clarifying the decision-making process of black box algorithms, their limitations highlight the need for inherently interpretable approaches. Specifically, in the context of medical imaging, there is a pressing need for approaches that not only offer transparent explanations but also align with clinical reasoning.

This study aims to develop and evaluate One Match (OM), a hypothesis-driven approach that builds on template matching to classify myocardial scarring while improving interpretability compared to conventional CNN-based methods. Unlike traditional approaches, this method uses labeled templates corresponding directly to known pathological features, offering a clear and interpretable rationale for each classification decision. This approach aims to maintain the high accuracy associated with advanced AI models while significantly enhancing interpretability, making the decision-making process understandable to clinicians. This study hypothesizes that a template matching-based system can achieve comparable or superior accuracy to traditional CNN methods while having the potential to increase trust in AI among clinical stakeholders and facilitate broader adoption in medical practice.

## Materials and methods

This study examines the application of One Match as an interpretable alternative to CNNs for myocardial scar classification. It also assesses the impact of support algorithms that aim to improve classification performance.

### Study design

Contrast-enhanced cardiac MRI data from 279 patients were obtained retrospectively from Buffalo General Hospital with approval from the State University of New York at Buffalo’s IRB. Data access for research was granted on 2/29/2016, and the dataset was accessed between 1/1/2017 and 5/31/2022. Due to the retrospective nature of the study, which involved only chart review, the IRB waived the requirement for participant consent. All data, which included protected health information, were handled in compliance with HIPAA regulations to ensure confidentiality and data security.

Of the 279 patients, 151 were included in the myocardial scarring (MS) group based on a documented history of coronary artery disease and visual evidence of myocardial scarring on cardiac MRI. The control group consisted of 128 patients without a history of significant coronary artery disease and no cardiac MRI evidence of myocardial scarring. The cohort’s average age was 61, with a gender distribution of 111 female and 168 male patients.

Data were acquired with a 1.5T GE Signa Excite scanner using the parameters noted in Table 1. The clinical imaging protocol for the acquisition is described in our previous work [14].

**Table 1:** Cardiac magnetic resonance imaging acquisition parameters.

Parameter	Value
Acquisition timing	7–10 minutes after gadolinium injection
Echo time	1.7–3.9 ms
Field of view	320–480 × 320–480 mm^2^
Flip angle	15°–25°
Pixel size	1.5625–1.875 × 1.5625–1.875 mm^2^
Number of slices	6–32
Repetition time	3.65–8.25 ms
Slice thickness	6–10 mm
Trigger delay	251–801 ms

Short axis cardiac MRI data were extracted from DICOM files, with optimal window levels applied from the DICOM tags during the extraction process. From the DICOMs, 4,193 contrast-enhanced cardiac MRI images were extracted at their native resolution of 256 × 256 pixels. Of these images, 2,347 were from patients with cardiac MRI evidence of myocardial scarring in their cardiac MRI, and 1,846 images were from patients with no cardiac MRI evidence of myocardial scarring.

### Lightweight preprocessing

Initial preprocessing was handled with Lightweight Preprocessing (LWP), a 5-step computational engineering approach for preparing contrast-enhanced cardiac MRI data for classification, using the same approach outlined in our previous work [15].

LWP leverages the near-central location of the heart in cardiac MRI, the circular shape of the left ventricle in short-axis images, and circle detection to isolate the left ventricle and surrounding myocardium (LVSM), the primary region assessed for myocardial scarring in cardiac MRI.

#### LWP Step 1.

Cardiac MRI imaging data was cropped down from its native 256 × 256-pixel resolution to 128 × 128 pixels corresponding to the center region of each image. This process is possible because of the near center focus of cardiac MRI data.

#### LWP Step 2.

To facilitate Step 3, image brightness is normalized to the same maximum brightness level.

#### LWP Step 3.

Hough Circle Transform is applied to detect the circular shape of the left ventricle that appears in cardiac MRI short axis images. Images were then cropped from 128 × 128 pixels to 80 × 80 pixels based on the coordinates of the detected circles.

#### LWP Step 4.

Images go through another brightness normalization to again facilitate HCT’s ability to detect circles in the images.

#### LWP Step 5.

A stricter set of circle detection criteria was used to remove images that did not contain the LVSM, while retaining those that do. Through this final step, the total number of images was reduced from 4,193 to 2,717. While LWP does not explicitly select slices, its filtering process determines which slices are retained by prioritizing images where the left ventricle and myocardium are more clearly detected.

### Dataset preparation

After preprocessing with LWP, data underwent a rigorous image quality assessment to remove any images that (1) did not contain the LVSM or (2) had artifacts that significantly impaired image quality, such as motion artifacts and signal distortions. After this assessment, the final dataset was composed of 835 images, 264 from patients identified as having myocardial scarring and 571 from patients without myocardial scarring.

The remaining 835 images were further split into a training dataset (599 images, 205 with myocardial scarring, 394 without myocardial scarring) and an external testing dataset (236 images, 59 with myocardial scarring, 177 without myocardial scarring). During this process, the image data were separated by patient, ensuring that each patient’s data did not overlap between the datasets.

### Algorithms

#### One Match.

One Match is a template matching-based algorithm that applies OpenCV’s Python implementation using the normalized correlation coefficient, which generates correlation scores that represent similarity between images, where −1 denotes the perfect inverse, 0 indicates no similarity, 1 represents a perfect match [16]. In preparation for One Match, data from the training portion of the dataset was separated into two template sets based on label, a positive template set for images from patients with myocardial scarring, and a negative template set for images from patients without myocardial scarring.

**Fig 1:**
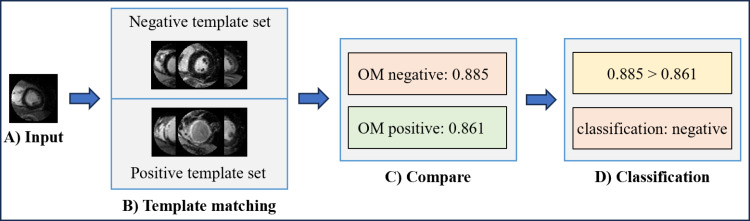
One Match (OM) workflow. A) Input image. B) The input image is matched with the positive and negative template sets, finding the highest correlation for each set. C) The highest correlation from each template set is then compared. D) The input image is classified based on which template set has the higher correlation. In this case, the negative template set has a higher correlation, so the classification is negative.

In One Match, each input image from the testing dataset is template matched against all images in the training dataset, producing correlation scores. Once this process is complete, One Match identifies the training dataset image with the highest correlation and determines whether it belongs to the positive template set or the negative template set. If the image with the highest correlation is from the positive template set, the classification is myocardial scarring. Fig 1 visualizes the workflow for OM.

#### Convolutional neural networks.

**Fig 2:**
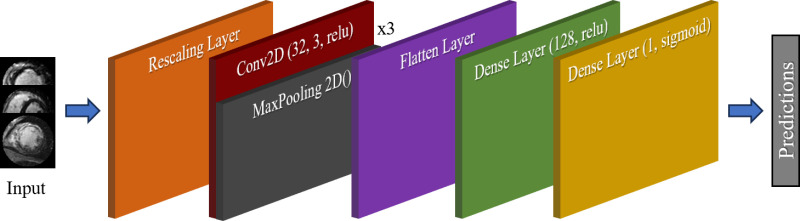
Architecture and workflow for sequential convolutional neural network (CNN). Lightweight Preprocessing was utilized to transform cardiac MRI data into the Input. The input data was then passed through the CNN to train it and make predictions about the presence of myocardial scarring in the images.

A sequential CNN was chosen for its computational efficiency and classification accuracy, enabling rapid iterative development while maintaining strong performance. Its lightweight design mirrors the efficiency, simplicity, and tunability of One Match, supporting a more direct and robust comparison by using the identical training and testing datasets. The CNN architecture, shown in Fig 2, used the Adam optimizer with a learning rate of 0.001, selected through prior experimentation, and was trained over 50 epochs with an 80/20 training/validation split. The ModelCheckpoint callback was also employed.

#### Autodidactic enhancement.

**Fig 3:**
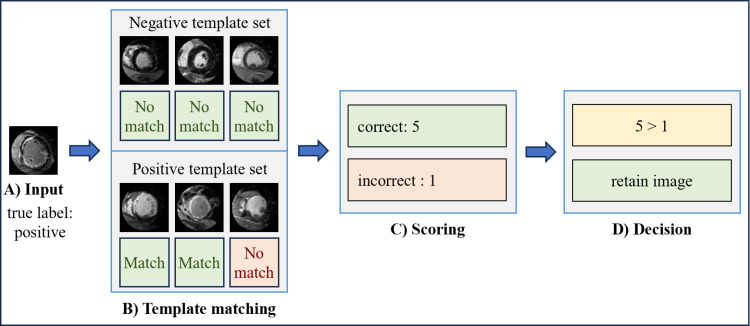
Autodidactic enhancement workflow. A) The true label of each input image is known. B) Each input is matched against the images in the positive and negative template sets, checking to see if it matches to the correct images and doesn’t match with the wrong ones. C) The number of correct and incorrect matches are summed. D) The decision is a simple majority decision. When the number of correct images is greater, the image is retained. Otherwise, the image is discarded.

The primary objective of Autodidactic Enhancement (AE) is to optimize the data used for MS classification. AE achieves this by applying Dynamic-threshold Template Matching (DTM), an algorithm developed in our lab’s previous work, which classifies images using template matching applied at a variable threshold [17]. Applying DTM, each image within the training dataset is template matched against all other images in the training dataset starting at a threshold of 0.9, excluding images from the same patient. This 0.9 threshold is important to this process as it is close to 1, a perfect match. This high starting mark allows DTM to focus on matches in the dataset starting at a high correlation. Since the correct labels are known for each image, this process generates a count of accurate and inaccurate matches for each one. An image is retained if it matches correctly more frequently than incorrectly; otherwise, it is discarded, eliminating less predictive images. This results in the removal of data in the training dataset that template matches incorrectly at high correlations with other data in the training dataset. This reduces the size of the template sets, accelerating classification time, and improves the quality of the template sets, leading to potential improvements in classification performance. The AE workflow is visualized in Fig 3.

#### Patient-level classifications.

Once single-image classifications are complete, patient-level classifications (PLCs) aggregate the results from all slices of a patient to produce one final, more reliable classification for that patient. The final decision for PLCs is based on a simple majority, with the confidence of the classifications used to break ties. This approach is particularly advantageous for medical images, where artifacts and individual patient physiological variability can lead to incorrect single-image classifications. Mathematically, if each individual classification has an accuracy of around 80%, performing multiple classifications and combining them increases the likelihood that the majority will be correct. By utilizing aggregated patient classifications with PLCs, diagnoses become more accurate and resilient to variations in individual images, reducing the impact of errors in individual slices. Further details on this method can be found in our previous work [15].

### Classification workflow

**Fig 4:**
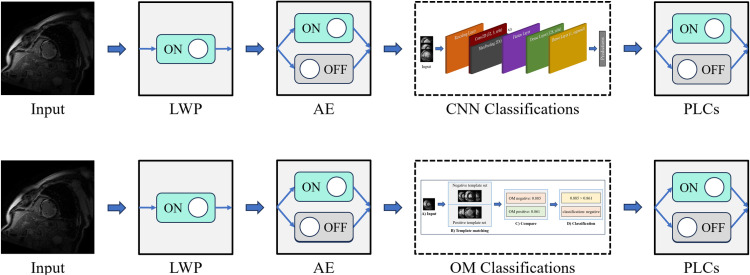
Classification workflow comparison for the sequential convolutional neural network (CNN) and One Match (OM). Input was first processed by Lightweight Preprocessing (LWP) and optionally processed with autodidactic enhancement (AE). Classifications were then performed with either the sequential CNN or with OM. Patient-level classifications (PLCs) were applied optionally as well.

Input data were processed using two major workflows, as shown in Fig 4. The first workflow employed a sequential CNN, which was run both with and without the use of AE and PLCs. The second workflow applied the OM approach, utilizing AE and PLCS with the same options for enhancement. This allowed for a direct comparison between the workflows with and without the enhancement techniques.

### Classification comparison

Classifications made by both the CNN and OM were analyzed with and without the presence of AE and PLCs. As CNN classifications were made using 20-fold cross-validation, the number of correct and incorrect classifications were compared fold-by-fold for each image to gauge its classification performance, with the median prediction used as a tiebreaker to decide the final classification made by the CNN in the case of a 10–10 tie.

### Classification traceback analysis

The combined classification information was also used to identify images that were misclassified across most folds by the CNN and misclassified by OM. For these images, the open decision-making of OM was leveraged to trace each classification back to the specific image in the template set that caused it. This information was used to create a visual comparison, which includes the misclassified image, the template image it matched to, and a blend of the two images showing their similarities and differences, facilitating the analysis of misclassifications.

### Data analysis

Classification performance was assessed through accuracy, sensitivity, specificity, precision, F1-score, and area under the receiver operating characteristic curve (AUROC). AUROC was calculated for OM by dividing the value of the best match for the positive template set by the sum of the best match for both template sets, creating a 0–1 value for each prediction. The resulting value is an estimation and is best used when comparing different versions of template matching. Of note, comparisons with PLC did not have AUROC as this method was incompatible. Additionally, the performance of the CNN was assessed based on a 20-fold cross-validation and reported as mean ± standard error.

All classifications were performed on an external testing dataset, untouched by the algorithms prior to classification. Additionally, data was carefully separated by patient to avoid dataset overlap.

### Statistical analysis

A one-sample t-test was used to compare the performance metrics (Accuracy, Sensitivity, Specificity, Precision, F1-score, and AUROC) of OM against the CNN. Paired t-tests were applied for comparisons between different CNN-based methods. A significance level of p < 0.05 was used to assess differences.

## Results

Classification performance was assessed for the CNN and OM, both with and without applying AE and PLCs, Table 2. AE impacted the CNN and OM differently, with the CNN decreasing in performance and OM increasing in performance. Incorporating PLCs reduced variability and improved performance across all combinations of methods and enhancements, with the highest performance achieved when OM was combined with AE. While baseline CNN performance and OM performance were similar, OM outperformed the CNN when AE and PLCs were applied.

**Table 2:** Classification performance for the convolutional neural network (CNN) and One Match (OM). Results for the CNN are shown as the average of a 20-fold cross-validation. Performance is reported with and without the application of the autodidactic enhancement (AE) algorithm and patient-level classifications (PLCs). The highest numerical value for each column is bolded. Values are reported as mean ± standard error for the CNN and value ± 0.000 for OM, which has no variability.

Method	Accuracy (%)	Sensitivity (%)	Specificity (%)	Precision (%)	F1-score (%)	AUROC
CNN	85.0 ± 0.317	82.5 ± 1.353	85.8 ± 0.571	66.2 ± 0.718	73.3 ± 0.581	**0.901 ± 0.002**
CNN + PLCs	89.1 ± 0.004	86.9 ± 0.015	90.0 ± 0.005	79.2 ± 0.008	82.7 ± 0.008	–
CNN + AE	82.4 ± 0.264	79.2 ± 1.386	83.5 ± 0.623	61.8 ± 0.581	69.2 ± 0.454	0.884 ± 0.002
CNN + AE + PLCs	86.9 ± 0.005	84.2 ± 0.016	88.0 ± 0.007	75.6 ± 0.001	79.4 ± 0.007	–
OM	85.6 ± 0.000	67.8 ± 0.000	91.5 ± 0.000	72.7 ± 0.000	70.2 ± 0.000	0.833 ± 0.000
OM + PLCs	93.0 ± 0.000	84.6 ± 0.000	**96.7 ± 0.000**	91.7 ± 0.000	88.0 ± 0.000	–
OM + AE	89.0 ± 0.000	76.3 ± 0.000	93.2 ± 0.000	78.9 ± 0.000	77.6 ± 0.000	0.859 ± 0.000
OM + AE + PLCs	**95.3 ± 0.000**	**92.3 ± 0.000**	**96.7 ± 0.000**	**92.3 ± 0.000**	**92.3 ± 0.000**	–

To evaluate the statistical significance of differences between the approaches, we conducted six comparisons, which are summarized in Table 3. Comparisons between OM variants were excluded due to the lack of variability, allowing for direct comparison of the values presented in Table 2.

**Table 3:** Comparison of performance metrics across various methods. Abbreviations: CNN = convolutional neural network, OM = One Match, AE = autodidactic enhancement, PLCs = patient-level classifications, Acc = Accuracy, Sens = Sensitivity, Spec = Specificity, Prec = Precision, F1 = F1-score, AUROC = Area Under the Receiver Operating Characteristic Curve. Note: AUROC for OM is an estimated measure best used for comparison between OM variants, comparisons to the CNN are included here for completeness.

Comparison	Metrics	p-value	Better method
CNN vs OM	Sens, F1, AUROC	< 0.001	CNN
	Acc	0.065	–
	Spec, Prec	< 0.001	OM
CNN vs CNN + AE	F1, AUROC, Acc	< 0.001	CNN
	Sens	0.046	CNN
	Spec	0.016	CNN
CNN + AE vs OM + AE	Acc, Spec, Prec, F1	< 0.001	OM + AE
	Sens	0.054	–
CNN + PLCs vs OM + PLCs	Acc, Spec, Prec, F1	< 0.001	OM + PLCs
	Sens	0.135	–
CNN + AE + PLCs vs OM + AE + PLCs	Acc, Sens, Spec, Prec, F1	< 0.001	OM + AE + PLCs
CNN + PLCs vs OM + AE + PLCs	Acc, Spec, Prec, F1	< 0.001	OM + AE + PLCs
	Sens	0.002	OM + AE + PLCs

**Fig 5:**
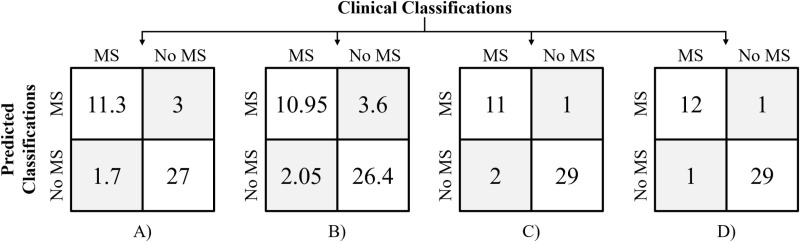
Confusion matrices for myocardial scarring (MS) classification with patient-level classifications. A) Cross-validated results for the convolutional neural network (CNN) without the autodidactic enhancement (AE). B) Cross-validated results for the CNN with the AE. C) Results for One Match (OM) without the AE. D) Results for OM with the AE. For the CNN, AE reduced both sensitivity and specificity, while it increased sensitivity for OM.

To further visualize PLC performance, confusion matrices were created, Fig 5. Confusion matrices for the CNN were calculated based on 20-fold cross-validation, with the average TPs, TNs, FPs, FNs reported, leading to some decimal results. OM outperformed the CNN both with and without AE, producing classifications using AE and PLCs where only 2 patients were classified incorrectly.

Further analysis focused on the classifications made by both algorithms. Classifications were broadly similar between the CNN and OM, with an average similarity of 90.5% between the CNN and OM with and without AE, showing the greatest similarity (91.5%) between the highest-performing versions of each method and the least (89.4%) between the lowest performing versions of each method, Table 4.

**Table 4:** Classification similarity comparison for the convolutional neural network (CNN) and One Match (OM).

Method	Similarity (%)
CNN vs OM	89.8
CNN + AE vs OM	89.4
CNN vs OM + AE	91.5
CNN + AE vs OM + AE	91.1
Average	90.5

To more closely examine the impact of AE, each of the 236 image classifications on the external testing dataset was examined for the CNN and OM with and without AE. Table 5 shows the number of correct and incorrect classifications made for each method both with and without AE, as well as the number of newly correct and incorrect classifications after AE was applied. As CNN classifications were made using 20-fold cross-validation, the number of correct and incorrect classifications were compared fold-by-fold for each image, with the median prediction used as a tie breaker in the case of a tie.

**Table 5:** Classification comparison for the convolutional neural network (CNN) and One Match (OM), with and without autodidactic enhancement (AE).

Method	Correct	Incorrect	New Correct	New Incorrect	Total Change
CNN	202	34	–	–	–
CNN + AE	195	41	4	11	−7
OM	202	34	–	–	–
OM + AE	210	26	8	0	+8

As shown in Table 5, AE negatively impacts the CNN, reducing the overall number of correct classifications by 7. In contrast, AE increased the number of correct classifications for OM by 8. Importantly, when examining the CNN classifications, there were 4 new correct classifications and 11 new incorrect classifications, highlighting the variability in CNN predictions.

**Fig 6:**
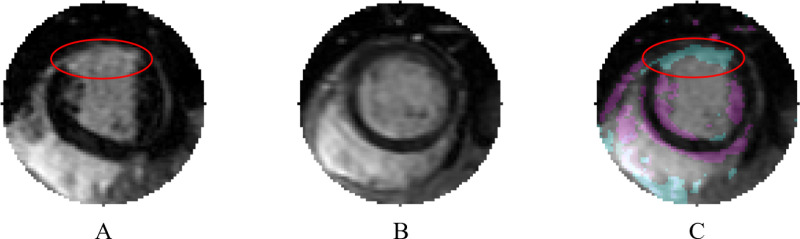
Image misclassified by One Match (OM). A) Misclassified image with scarring indicated by red oval. B) Template set image that had the highest correlation. C) Blend of part A and part B, cyan indicates regions present in A but not in B, and magenta indicates regions present in B but not in A. The area of scarring, highlighted by a red oval, appears in cyan. Even with this scar present, the 0.841 correlation between part A and part B resulted in part A being classified as not having scar.

**Fig 7:**
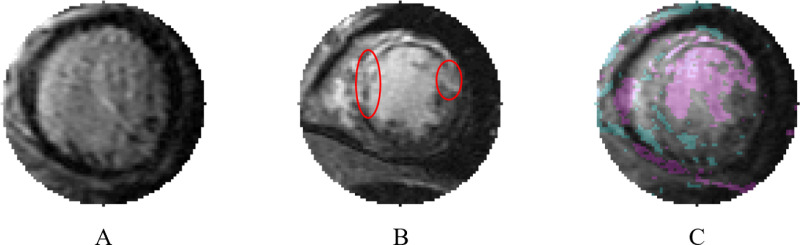
Image without scarring misclassified by One Match (OM). A) Misclassified image without myocardial scarring. B) Template image that had the highest correlation. C) Blend of part A and part B, with cyan indicating which regions present in A not present in B and magenta indicating the opposite. Despite what might be considered drastic differences by a human clinical observer, these two images yielded a correlation score of 0.826.

When examining both algorithms with and without AE, 20 images were misclassified by both methods. Using OM’s interpretable decision-making, error traceback was performed for these misclassifications, facilitating the creation of the visual comparisons shown in Figs 6 and 7.

In Fig 6, part A shows a misclassified image with myocardial scarring highlighted by a red oval, yet the algorithm classified it as not having scarring. Part B displays the template image with the highest correlation (most similarity) to the misclassified image. Although structurally like part A, part B does not exhibit myocardial scarring. Part C presents a blend of part A and part B, with cyan highlighting areas present in image A but not in image B and magenta indicating areas present in image B but not in image A. Part of the regions highlighted in cyan corresponds to the area of the scar (red oval). Despite the presence of this scar, the overall strong similarities between the images account for the template matching correlation of 0.841.

Fig 7 illustrates a case where an image without myocardial scarring is incorrectly classified as having scarring. Like Fig 6, part A shows the misclassified image, part B displays the image with the highest template-matched correlation to the image in part A, and part C is a blended image of parts A and B. The blend highlights areas with significant differences between the images, with cyan indicating areas more prominent in part A and magenta indicating areas more prominent in part B. Areas of myocardial scarring in part B are marked by red ovals. These areas obscure the myocardium around the left ventricle, causing the left ventricle and right ventricle to appear as a single structure. In contrast, part A shows a large left ventricle with and a myocardium with no visible myocardial scarring. Despite these differences, template matching yielded a correlation of 0.826 between the images, with part C demonstrating both the differences and strong similarities between the part A and part B.

## Discussion

This study demonstrates the efficacy of OM in producing high-accuracy MS classifications. OM utilizing both AE and PLCs, outperformed the CNN. While AE benefited OM, it had a detrimental effect on the CNN, suggesting that AE’s advantage may be limited to specific applications. In contrast, PLCs consistently enhanced performance for both OM and the CNN. Despite methodological differences, OM and the CNN produced very similar classifications, indicating that both methods effectively capture the same underlying patterns in the data and underscoring their reliability and robustness. Error traceback analysis with OM facilitated the examination of misclassifications, helping to identify and understand the algorithm’s weaknesses and underscoring the importance of interpretable decision-making in algorithm development. Overall, combining of OM with AE and PLCs improved myocardial scar classification, suggesting that this approach has potential to serve as an alternative to CNNs for pathological feature classification.

OM demonstrates substantial capability in accurately classifying myocardial scarring. In addition to performance, another key advantage is its interpretability, which fosters trust and is essential for adoption [18,19]. In contrast, reliance on black-box algorithms poses potential risk of harm to stakeholders, particularly for the high-stakes decisions made in clinical settings [20–22].

Error traceback is a powerful tool for refining algorithm development, providing insights into areas where improvements can be made. This feature allowed us to identify two significant limitations of OM.

The first limitation identified was OM’s reliance on a fixed-size area to classify scarring around the left ventricle. Since ventricles vary greatly in size between patients, different portions of this fixed area contain relevant information for each individual. As a result, the algorithm’s focus may shift toward non-scar regions, increasing the risk of misclassification. This suggests that the algorithm could be improved by dynamically adjusting to the size of the left ventricle in the image space, either normalizing or removing non-relevant areas to better target the region of interest. Such adjustments could enhance classification accuracy and improve the robustness of the algorithm.

The second limitation identified through error traceback was the size and composition of the dataset. There was a numerical imbalance between the positive and negative template sets, making it more challenging to accurately classify positive images. For instance, in Fig 6, despite the clear presence of scar, no sufficiently similar image could not be found in the positive template set. The composition of the dataset also lacked variety, as seen with the issue raised in Fig 7, where the algorithm was unable to find a comparable image in the negative template set. Expanding the dataset, particularly by balancing the two classes and increasing its variety, would likely improve the algorithm’s performance and generalizability. This suggests that future work should aim to acquire a more diverse, balanced, and comprehensive dataset to enhance classification robustness.

Error traceback with OM revealed not only weaknesses but also potential paths for improvement. By implementing these improvements, classification performance could potentially be enhanced for both OM and the CNN. This underscores the importance of inherently interpretable algorithms, as they facilitate the identification of issues and guide the development of solutions.

Outside of error traceback, data exclusion adversely affected the CNN’s performance, highlighting an important limitation of AE. While AE helped OM generate more accurate template matches, it inadvertently decreased CNN accuracy. This discrepancy suggests that the CNN was making productive use of the excluded data to improve its performance, meaning that the loss of this data represented a critical loss of information. Future refinements could involve strategies to mitigate performance reductions due to data loss, but also could be aimed at enhancing OMs ability to utilize a broader range of data to improve classification performance.

Overall, the insights gained from error traceback and the highlighted limitations emphasize the potential for OM to evolve into an even more robust tool for myocardial scar classification. By addressing these areas for improvement—particularly adapting to patient variability and expanding dataset diversity—OM’s potential for enhanced performance could be realized. Additionally, leveraging its inherently interpretable nature to guide algorithmic development underscores its value not just for OM but for future advancements in medical image classification.

## Conclusions

This study demonstrates the potential value of One Match as a high-accuracy alternative to CNNs for pathological feature classification. Through its interpretable operation, One Match facilitates valuable introspection which has promise in informing algorithmic development. Future research should focus on developing interpretable approaches that improve performance without compromising the trust of stakeholders.
